# Meta-Learning Task Relations for Ensemble-Based Temporal Domain Generalization in Sensor Data Forecasting

**DOI:** 10.3390/s25144434

**Published:** 2025-07-16

**Authors:** Liang Zhang, Jiayi Liu, Bo Jin, Xiaopeng Wei

**Affiliations:** 1 School of Computer Science and Technology, Dalian University of Technology, Dalian 116024, China; liangzhang@dlut.edu.cn (L.Z.); liujiayikm@mail.dlut.edu.cn (J.L.); 2 Key Laboratory of Social Computing and Cognitive Intelligence, Ministry of Education, Dalian 116024, China; 3 School of Innovation and Entrepreneurship, Dalian University of Technology, Dalian 116024, China

**Keywords:** temporal domain generalization, time series forecasting, meta-learning

## Abstract

Temporal domain generalization is crucial for the temporal forecasting of sensor data due to the non-stationary and evolving nature of most sensor-generated time series. However, temporal dynamics vary in scale, semantics, and structure, leading to distribution shifts that a single model cannot easily generalize over. Additionally, conflicts between temporal domain-specific patterns and limited model capacity make it difficult to learn shared parameters that work universally. To address this challenge, we propose an ensemble learning framework that leverages multiple domain-specific models to improve temporal domain generalization for sensor data forecasting. We first segment the original sensor time series into distinct temporal tasks to better handle the distribution shifts inherent in sensor measurements. A meta-learning strategy is then applied to extract shared representations across these tasks. Specifically, during meta-training, a recurrent encoder combined with variational inference captures contextual information for each task, which is used to generate task-specific model parameters. Relationships among tasks are modeled via a self-attention mechanism. For each query, the prediction results are adaptively reweighted based on all previously learned models. At inference, predictions are directly generated through the learned ensemble mechanism without additional tuning. Extensive experiments on public sensor datasets demonstrate that our method significantly enhances the generalization performance in forecasting across unseen sensor segments.

## 1. Introduction

Sensor data forecasting plays a critical role in a wide range of real-world applications, serving as the foundation for intelligent decision-making and proactive system control. It has been widely adopted in diverse domains, including electricity consumption forecasting [1], traffic prediction [2], and weather forecasting [3]. Given the increasing scale, heterogeneity, and temporal dynamics of sensor data, developing robust forecasting models has become a fundamental challenge in the era of intelligent systems. Due to the non-stationary nature of the real-world streaming environment [4], the assumption that the data is independent and identically distributed (i.i.d.) cannot be satisfied [5]. This violation severely limits the generalization ability of traditional machine learning models, which tend to suffer significant performance degradation when confronted with temporal distribution shifts or previously unseen patterns.

To overcome these challenges, domain generalization (DG) techniques have been proposed to learn domain-invariant representations and forecasting models that maintain robustness across temporal domains [6,7,8], without requiring access to data from future tasks or environments during training. Existing approaches to temporal domain generalization typically rely on either data augmentation strategies—such as constructing diverse training environments to simulate distributional variations [9]—or domain-agnostic representation learning techniques aimed at extracting invariant features [10]. However, these methods often learn a single forecasting model. In practice, the evolving and dynamic characteristics of sensor data introduce both marginal and conditional distribution changes over time, making it difficult for a single, capacity-limited model trained on historical data to maintain effectiveness in future scenarios.

As highlighted in the model DRAIN [8], effective temporal forecasting not only requires that the data dynamics are modeled, but also requires the dynamic= adaptation of the forecasting model itself over time. DRAIN addresses this by using a Long Short-Term Memory (LSTM) network [11] to capture model dynamics and generate task-specific parameters for future prediction. Although this strategy leads to improved performance, it still suffers from limitations in capturing long-range dependencies and may underperform when historical dynamics exert delayed or accumulated influences. Furthermore, due to the non-stationary nature of temporal data, the distribution of model dynamics itself evolves over time, making it difficult for a single model to generalize across diverse and shifting forecasting tasks. This challenge motivates the use of ensemble-based strategies, where multiple specialized models can be integrated to cover a broader spectrum of temporal variations. Such ensemble learning approaches have demonstrated their effectiveness in various domains, as evidenced by Exemplar-SVMs [12], GMOE [13], and $D^{3}$G [14], highlighting their potential to enhance generalization by leveraging model diversity.

In this paper, we focus on the challenging problem of sensor data forecasting under temporal domain shifts. Unlike prior approaches that rely on fixed weighting schemes [12] or data-driven weight calculation based on auxiliary meta data [14], we propose METDG, a novel framework that automatically models the dynamic relationships among domain-specific forecasting models tailored to non-stationary sensor environments. Specifically, when given a time series from sensor data, we first partition it into semantically meaningful temporal segments—such as weeks, months, quarters, or years—based on domain knowledge or expert heuristics. These segments are treated as distinct tasks to reflect potential distributional shifts over time. To capture commonalities and distinctions across tasks, we adopt a meta-learning strategy that learns to generalize from shared temporal patterns. During the meta-training stage, we introduce a variational inference network to encode the support set of each task into a rich contextual representation. This embedding captures the underlying task-specific dynamics and is fed into a lightweight decoder (e.g., MLP) to generate the parameters of the forecasting model for that task. Importantly, to uncover global inter-task relationships, we leverage a Transformer-based encoder [15] to model the long-range dependencies among tasks and generate relationship-aware model parameters. In the inference phase, each sample from the query set is evaluated using all task-specific models in an ensemble manner, enabling the system to adaptively integrate multiple temporal perspectives for robust prediction. This design allows the model ensemble to dynamically respond to evolving sensor data characteristics, thereby enhancing generalization across unseen temporal domains. Our main contributions are listed as follows:

- We propose METDG, a novel ensemble-based meta-learning framework that segments sensor time series into semantically meaningful tasks and learns task-specific forecasting models via variational inference. A Transformer-based encoder is further leveraged to capture long-range inter-task relationships and generate relationship-aware model parameters.
- We design an adaptive ensemble inference mechanism that evaluates each query using all task-specific models, enabling the dynamic integration of temporal perspectives.
- We conduct extensive experiments on different real-world temporal forecasting tasks to show that our method outperforms state-of-the-art time series prediction methods and temporal domain generalization methods.

## 2. Related Work

**Time Series Forecasting.** Time series forecasting refers to predicting future changes based on historical data. Early researchers used statistical learning methods for forecasting, such as exponential smoothing and ARIMA models [16], Kalman Filter [17], and Linear State-Space models  [18]. With the advent of deep learning techniques, deep neural networks have increasingly been utilized for time series forecasting, owing to their exceptional ability to model complex nonlinear relationships. To capture the dependencies between time steps, researchers introduced recurrent neural network (RNN) methods, e.g., the Long Short-Term Memory network (LSTM) and Gated Recurrent Unit network (GRU), and Autoregressive Recurrent Neural Network methods, e.g., DeepAR [19]. For example, Salinas et al. [19] proposed an autoregressive recurrent neural network for probabilistic forecasting. In recent years, with the tremendous success of the Transformer model [15] in capturing global temporal patterns, researchers have also incorporated them into time series forecasting tasks, proposing algorithms such as Informer [1], FEDformer [20], Non-Stationary Transformer [21], DLiner [22], and Timer [23]. For instance, given the non-stationary nature of many time series (e.g., sensor data), Liu et al. proposed a non-stationary Transformer to mitigate the over-stationarization issue in forecasting tasks. The experimental results on sensor datasets such as Electricity and Traffic validate the effectiveness of the proposed model; Liu et al. [23] also developed a foundation model tailored for general-purpose time series modeling. In addition, multivariate times series prediction methods have also been focusing on variable relationship modeling, together with forecasting [3,24]. For instance, Cai et al. [3] proposed learning multi-scale inter-series correlations for multivariate time series forecasting. Despite recent advances, time series forecasting remains vulnerable to distribution drift, which poses a major challenge to model generalization and robustness across varying temporal domains [25,26]. In addition, recent works have begun to exploit graph structures to model complex dependencies in forecasting. For example, Yang et al. [27] proposed DEST-GNN, a double-explored spatio-temporal GNN for multi-site intra-hour photovoltaic (PV) power forecasting. Similarly, Fu et al. [28] introduced SDR-GNN, which constructs an utterance-level interaction graph in conversational data and uses spectral-domain reconstruction (multi-frequency aggregation) to recover incomplete multimodal features for emotion recognition. Hou et al. [29] further developed a parallel multi-scale dynamic GNN (PMEDGN) that learns dynamic graph structures at multiple time resolutions in parallel, capturing evolving inter-variable interactions in multivariate time series forecasting. These dynamic graph-based approaches suggest that incorporating graph neural network structures is beneficial for modeling the spatial relationships between sensors in time series forecasting tasks.

**Temporal Domain Generalization.** Typical machine learning methods assume that the training data and test data follow the same probability distribution. However, the non-stationary nature of sensor data violates the i.i.d. assumption [21,30]. Domain generalization learning aims to learn robust models without using test domain data, making it appropriate for future time series inference. Therefore, temporal domain generalization has attracted researchers’ attention. In general, these methods can be grouped into three categories [8]. First, data manipulation [9], which builds complex data training environments via data generation or data augmentation. Second, representation learning [10], which aims to learn domain-invariant representations via feature alignment or invariant risk minimization. For instance, Fan et al. [10] proposed to separately learn the distribution of input and output space, which would naturally capture the distribution difference of the two spaces. Third, learning strategy [6,8], which focuses on exploiting the ensemble learning strategy [6], meta-learning strategy [6,31,32], and gradient interpolation strategy [7]. For example, Li et al. [6] introduced a lifelong learning mechanism in sequential domain generalization, while Bai et al. [8] proposed using a sequential model to adaptively learn the temporal drift and leverage the learned sequential pattern to predict the model status in the future domain. However, existing methods either use a single model or employ multiple models, without considering the global dynamic relationships between models or assigning appropriate weights.

## 3. Methodology

### 3.1. Problem Formulation

In a sensor data forecasting task, we are provided with historical multivariate time series data, denoted as $X\in R^{H\times N}$, where *H* is the length of the historical window and *N* is the number of sensor channels. The objective is to learn a model that accurately predicts the future sensor readings $Y\in R^{F\times N}$ over the following *F* time steps. However, sensor data in real-world applications often exhibit temporal distribution shifts, where the underlying data distribution changes over time. These evolving patterns violate the stationary assumption commonly made by standard forecasting models, resulting in a degraded performance when deployed in unseen or shifted temporal domains. Our goal is to develop a forecasting model that is robust to such distributional shifts, thereby maintaining low prediction error on test data drawn from future, potentially shifted time periods.

During the training process, we divide the historical training data into *T* different segments (i.e., domains) $D_{1},D_{2},\ldots ,D_{T}$, where each segment (i.e., domain) contains *n* samples $D_{t}={\left\{\left(X_{i},Y_{i}\right)\right\}}_{i=1}^{n}$. We assume that the data within each domain follow the same distribution, while the distributions differ across domains. For each domain *t*, a neural network model $g_{\Phi_{t}}$ can be learned by minimizing the loss function $\sum_{i=1}^{n}l\left(g_{\Phi_{t}}\left(X_{i}\right),Y_{i}\right)$, where $\Phi_{t}$ represents the parameters of the neural network. Our objective is to generate model parameters for a new, unseen domain $D_{T+1}$ based on the data from the existing domains $D_{1},D_{2},\ldots ,D_{T}$, such that the empirical risk for $D_{T+1}$ is minimized.

### 3.2. Model Architecture

In this section, we present the architecture of our proposed METDG model. To effectively capture temporal domain-specific characteristics and enhance the model’s generalization capability across temporally shifting distributions, we adopt a Bayesian meta-learning framework. Unlike conventional meta-learning approaches that learn deterministic task-specific representations, Bayesian meta-learning [33] models the uncertainty and variability across tasks (i.e., domains) by learning a distribution over model parameters. This probabilistic formulation allows the model to better adapt to new, unseen domains by leveraging prior knowledge while accounting for domain-specific deviations. In our context, each domain corresponds to a time segment of sensor data with distinct temporal patterns; thus, modeling such domain-level uncertainty is crucial for robust forecasting under distributional shifts.

Following the Bayesian meta-learning framework, our approach consists of two main stages: meta-training and meta-testing. For each domain $D_{t}$, we partition the data into a support set $D_{t}^{Su}$ and a query set $D_{t}^{Qu}$, i.e., $D_{t}=\left\{D_{t}^{Su},D_{t}^{Qu}\right\}$. In the meta-training phase, our goal is to train an encoder to extract representations of the domains. In the meta-testing phase, we use the extracted representations to generate neural network parameters corresponding to each domain, and evaluate them using meta-testing data, calculate the loss, and perform backpropagation to train the parameters of the encoder and decoder. Assuming that $P(D_{t})$ is the probability density function associated with $D_{t}$, the process of meta-learning can be formally expressed as follows:

(1) $$\underset{\theta }{argmin}\sum_{t=1}^{T}E\left(l\left(g_{\Phi_{t}}\left(X_{t}^{Qu}\right),Y_{t}^{Qu}\right)\right),\Phi_{t}=f_{\theta }\left(D_{t}^{Su}\right)$$

where $f_{\theta }$ represents the encoder parameterized by θ, which extracts domain representations from the support set, and $g_{\Phi_{t}}$ represents the inference network parameterized by $\Phi_{t}$. According to the theory of Bayeasin meta-learning, it is assumed that each domain has a latent variable z, which represents the domain and leads to variation in domain distribution. By learning a representation of this latent variable, we can effectively model the distributional differences among domains, thereby enhancing the model’s adaptability and generalization abilities. To infer the latent variable z, we utilize the support set of each domain as contextual input. Specifically, an encoder network processes the support set to approximate the posterior distribution over z, capturing rich domain-specific information. This latent representation is then passed through a decoder, which generates the parameters for a domain-specific inference network. Finally, the query input *X* from the same domain is fed into the generated inference network to produce prediction *Y*. This entire process can be naturally formulated within a probabilistic framework, where both the uncertainty in domain representations and the domain-specific adaptation are explicitly modeled.

The probabilistic formulation of this process is provided as follows:

(2) $$\begin{array}{cc} P(D_{1:T}^{Su}, & X_{1:T},Y_{1:T},z_{1:T},\Phi_{1:T})=P\left(D_{1:T}^{Su},z_{1:T}\right)P\left(X_{1:T},Y_{1:T},\Phi_{1:T}|D_{1:T}^{Su},z_{1:T}\right). \end{array}$$

In summary, the architecture of our model can be conceptually divided into two main components: (1) domain representation learning and (2) parameter generation for domain-specific inference networks. In the first stage, we extract domain-specific representations from the support set. Specifically, the input time series from each support set is processed by a diffusion convolutional GRU-based encoder, which captures both temporal dependencies and spatial correlations among sensors. Note that the encoder can also employ GRU or LSTM for sensor prediction tasks where there are no spatial dependencies among sensors. To further model inter-domain dependencies, a self-attention mechanism is employed over the encoded features, enabling the model to learn rich and informative domain relationships. This results in a set of contextualized domain representations that reflect both intra-domain characteristics and cross-domain interactions. In the second stage, these domain representations are passed through a lightweight decoder to generate the parameters of a domain-specific inference network. This inference network is instantiated using the decoded parameters and then applied to the query set: it takes input *X* and produces predictions *Y* tailored to the specific domain characteristics. This two-stage design allows the model to dynamically adapt to unseen domains by leveraging learned cross-domain knowledge and ensures robust forecasting performance under temporal distribution shifts.

**Figure 1:**
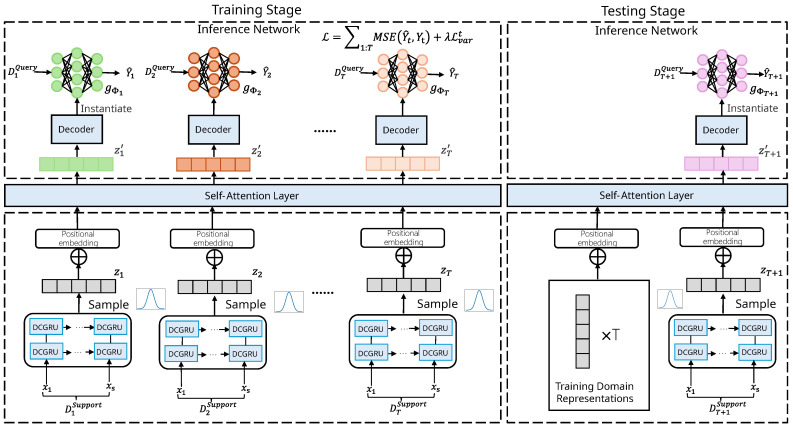
The overall architecture of the METDG model, which contains three parts: (1) a GRU-based encoder; (2) a domain relation modeling module based on a self-attention mechanism; (3) an inference network. The left part of the architecture illustrates the training phase, while the right part corresponds to the testing phase.

The overall framework is illustrated in Figure 1. In the following sections, we provide a detailed description of the core modules of our model: the GRU-based encoder and the self-attention mechanism for learning relational domain representations, and the inference network for generating domain-specific parameters.

#### 3.2.1. DCGRU-Based Encoder

In the first component of our formulation, the objective is to extract domain-specific feature representations from the support set data, denoted as $P(z_{1:T}|D_{1:T}^{Su})$. To achieve this, we adopt the diffusion convolutional gated recurrent unit (DCGRU) architecture, following the approach of Liu et al. [24]. DCGRU is designed to effectively model both the temporal and spatial dependencies inherent in the sensor data of each domain. Specifically, it integrates diffusion convolution to capture the spatial correlations among sensors, and employs the Gated Recurrent Unit (GRU) to model temporal dynamics. This hybrid architecture enables the encoder to produce expressive and informative representation vectors for each domain. The operations of DCGRU are defined as follows:

(3) $$\begin{array}{cc} \; & R_{t^{\prime }}=sigmoid(W_{R★A}[X_{t}\left|\right|H_{t-1}]+b_{R}),\\ & C_{t^{\prime }}=tanh(W_{C★A}[X_{t}\left|\right|R_{t}⊙H_{t-1}]+b_{C}),\\ & U_{t^{\prime }}=sigmoid(W_{U★A}[X_{t}\left|\right|H_{t-1}]+b_{U}),\\ & H_{t^{\prime }}=U_{t}⊙H_{t-1}+\left(1-U_{t}\right)⊙C_{t}, \end{array}$$

where *A* is the adjacency matrix representing the connectivity between sensors. The graph convolution is computed as follows:

(4) $$\begin{array}{c} W_{Q★A}X=\Sigma_{k=0}^{K}\left(w_{k,1}^{Q}{\left(D_{O}^{-1}A\right)}^{k}+w_{k,2}^{Q}{\left(D_{I}^{-1}A\right)}^{k}\right)X, \end{array}$$

where $D_{O}^{-1}$ and $D_{I}^{-1}$ are the out-degree and in-degree matrices, respectively, and $w_{k,1}^{Q}$ and $w_{k,2}^{Q}$ are the trainable model parameters. The diffusion feature *K* is a hyperparameter.

For each domain, the encoder sequentially takes support set data $D_{t}^{Su}$ as inputs, and outputs the distribution of the domain representation. Here, we assume that the data from all domains follow a normal distribution. The optimization process of the encoder can be carried out using the variational inference method. This approach is particularly suitable for extracting features from different domains. By introducing a domain-level latent variable and inferring its posterior from observed data, variational inference provides a principled way to capture cross-domain variability. In contrast, other probabilistic approaches, such as Bayesian neural networks, primarily model parameter uncertainty and lack an explicit mechanism to represent domain-specific structure. Therefore, variational inference is adopted to model uncertainty at the representation level, which better aligns with the objectives of our meta-learning framework. According to the theory of variational inference, the posterior distribution $q(z_{1:T}|D_{1:T}^{Su})$ has the following variational lower bound(ELBO):

(5) $$\begin{array}{c} L_{t}^{var}=E\left[logp\left(D_{t}^{Su}|z_{t}\right)\right]-D_{KL}(q\left(z_{t}|D_{t}^{Su}\right)||p\left(z_{t}\right)) \end{array}$$

where $p(D_{t}^{Su}|z_{t})$ represents the reconstruction of $D_{t}^{Su}$, and $p(z_{t})$ denotes the prior distribution, which is assumed to be a standard normal distribution. $D_{KL}\left(\cdot ||\cdot \right)$ means the KL divergence between the posterior distribution and the prior distribution.

It is worth noting that, during training, the data is first partitioned into *T* distinct domains in chronological order. For feature extraction, an encoder with shared parameters is applied concurrently across all domains. In other words, the feature distributions for all domains are extracted simultaneously. To incorporate stochasticity into the domain generalization process, the encoder does not directly output domain representations. Instead, after the support set passes through two DCGRU layers, the resulting features are fed into a fully connected layer that produces the mean and log-variance of a Gaussian distribution. The representation for each domain is then obtained through sampling from its corresponding distribution.

#### 3.2.2. Domain Relation Modeling

After extracting features from the *T* domains using a shared encoder, a self-attention mechanism is employed to model inter-domain relationships, thereby enabling dynamic domain generalization. Let $Z=\{z_{1},z_{2},\ldots ,z_{T}\}$ denote the representations of the *T* training domains, where each $z_{t}$ is a *k*-dimensional tensor. The process of modeling domain relationships is described as follows:

First, positional embeddings are applied to incorporate temporal or sequential information:

(6) $$\begin{array}{c} Z=Z⊕P. \end{array}$$

It is important to note that a fixed absolute positional encoding scheme is adopted. For each representation vector $z_{t}$, the corresponding positional encoding vector is defined as follows:

(7) $$\begin{array}{cc} p_{t,2i} & =sin(k/10000^{\frac{2i}{d}}),\\ p_{t,2i+1} & =cos(k/10000^{\frac{2i+1}{d}}). \end{array}$$

After obtaining the positional embeddings, the matrix Z is projected through three learnable weight matrices—$W^{Q},W^{K}$, and $W^{V}$—to generate the query, key, and value embeddings Q, K, and V, respectively:

(8) $$\begin{array}{c} Q=ZW^{Q},K=ZW^{K},V=ZW^{V}. \end{array}$$

Next, the self-attention mechanism is applied to Q, K, and V to compute attention scores and aggregate information, resulting in the following domain relation representations:

(9) $$\begin{array}{c} Z=SA\left(Q,K,V\right)=softmax\left(\frac{{\mathrm{QK}}^{T}}{\sqrt{d_{q}}}\right)V. \end{array}$$

Since the encoder outputs distributions over domain representations, sampling must be performed from these distributions before feeding them into the self-attention layer. The domain relation modeling process can be formally defined as follows:

(10) $$\begin{array}{c} z_{t}^{\prime }=\frac{\sum_{s=1}^{T}d_{s,t}z_{t}}{\sum_{s=1}^{T}d_{s,t}},z_{t}=f\left(D_{t}^{Su}\right). \end{array}$$

The self-attention mechanism plays a central role in modeling the global dynamic relationships between domains. Specifically, it allows each domain representation to attend to all other domains in the input sequence, regardless of their temporal proximity. By computing pairwise attention scores based on learned similarities, the model is able to capture long-range dependencies and latent correlations across domains. This enables it to dynamically weigh the influence of each domain when generating the task-specific representation, thus reflecting broader temporal trends, recurrent patterns, or shared dynamics.

#### 3.2.3. Decoder and Inference Network

After obtaining the related domain representations, they are passed into a decoder to generate the corresponding parameters for each domain. These parameters are then used to instantiate an inference network for each domain. In this work, a two-layer multilayer perceptron (MLP) is adopted as the inference network. The query set data is subsequently fed into the respective inference networks to produce the prediction results, followed by the computation of the supervised loss:

(11) $$\begin{array}{cc} L_{t}^{sup} & =MSE(g_{\Phi_{t}}\left(X_{t}^{Qu}\right),Y_{t}^{Qu}), \end{array}$$

where $g_{\Phi_{t}}$ denotes the inference network parameterized by $\Phi_{t}$, and $X_{t}^{Qu}$ and $Y_{t}^{Qu}$ represent the input features and corresponding labels of the query set for domain *t*, respectively.

To achieve effective training, we aim to minimize the supervised loss while simultaneously maximizing the variational lower bound. By combining these two objectives, the overall loss function used during optimization is defined as follows:

(12) $$\begin{array}{c} L=\sum_{t=1}^{T}\left(L_{t}^{sup}+\lambda L_{t}^{var}\right), \end{array}$$

where λ is used to balance supervised loss and variational loss.

### 3.3. Model Training and Testing Process

In this section, we present the training and testing procedures of METDG. The overall workflow of the proposed model consists of two main stages: training and testing. During the training stage, the model learns transferable knowledge from multiple known domains by encoding their support sets, modeling inter-domain relationships via self-attention, and using a decoder to generate domain-specific inference networks. The model is optimized by computing prediction losses on query sets across domains. During the testing stage, the model uses the support set of an unseen domain to generate its representation, reweight it using stored training domain representations, and constructs an inference network for final prediction on the test query set.

**During the training stage**, we empirically divide the training data into *T* distinct domains. Each domain’s data is further split into a support set and query set. The support set from each domain is sequentially fed into the encoder to produce a distribution over domain representations. We then sample the representations from these distributions and apply a self-attention mechanism to model the relationships among the domains. The resulting representations are passed through a decoder, which generates the parameters required to instantiate the domain-specific inference networks. Specifically, we use a multi-layer perceptron (MLP) as the inference network architecture. The query set is then fed into the corresponding inference network to produce predictions ${\hat{Y}}_{1:T}$. For each domain, we compute the loss using Equation (11), sum the losses across all domains to obtain the total training loss, and update model parameters via backpropagation. After training, we store the learned representations of all domains for use during the testing phase. The complete training procedure is summarized in Algorithm 1.

**Algorithm 1** Training Procedure**Require:** Training domain data $D_{1}$,…,$D_{T}$, meta split ratio ζ, learning rate η, initialized      encoder $f_{\theta }$      Initialize all learnable parameters      Split ${\left\{D_{t}\right\}}_{t=1}^{T}$ into $D_{t}^{Su}$ and $D_{t}^{Qu}$ using split ratio ζ      **while** not converge **do**           **for**
${\left\{\left(X,Y,t\right)\right\}}_{t=1}^{T}$ in ${\left\{D_{t}^{Qu}\right\}}_{t=1}^{T}$
**do**               **for**
t=0 to *T*
**do**                    Sample $z_{t}∼$
$q(z_{t}|D_{t}^{Su},\theta )$                    Compute log likelihood using Equation (2)               **end for**               $Z^{\prime }=SA\left(Z\right)$               ${\left\{\Phi_{t}\right\}}_{t=0}^{T}$ = $F(z_{t}^{\prime })$               ${\left\{{\hat{Y}}_{t}\right\}}_{t=1}^{T}$ = ${\left\{g_{\Phi_{t}}\left(X_{t}\right)\right\}}_{t=1}^{T}$           **end for**           Calculate supervised loss $L_{t}^{sup}$ and Consistency loss $L_{t}^{var}$ with ${\left\{{\hat{Y}}_{t}\right\}}_{t=1}^{T}$ and ${\left\{\left(Y_{t}\right)\right\}}_{t=0}^{T}$           $L=L_{t}^{sup}+\lambda L_{t}^{var}$           Update learnable parameters with learning rate η      **end while**

**During the testing stage**, the unseen test domain is similarly divided into a support set and query set. The support set is used to generate the representation of the test domain. Subsequently, a self-attention mechanism is employed to extract relevant information by attending to the saved training domain representations. Using the updated test domain representation, the decoder generates parameters to instantiate the corresponding inference network. The query set is then fed into this inference network to produce the final prediction results. The complete testing procedure is outlined in Algorithm 2.

**Algorithm 2** Testing Procedure**Require:** Training domain representation $z_{1}$,…,$z_{T}$,Testing domain data $D_{T+1}$, meta split      ratio ζ      Split $D_{T+1}$ into $D_{T+1}^{Su}$ and $D_{T+1}^{Qu}$ using split ratio ζ      Sample $z_{T+1}∼$$q(z_{T+1}|D_{T+1}^{Su},\theta )$      $z_{T+1}^{\prime }=SA\left(z_{1:T},z_{T+1}\right)$      $\Phi_{T+1}$ = $F(z_{T+1}^{\prime })$      **for** each example (X,Y) in $D_{T+1}^{Qu}$ **do**           ${\hat{Y}}_{T+1}$ = $g_{\Phi_{T+1}}\left(X_{T+1}\right)$           Calculate MSE loss with $\hat{Y}$ and **Y**      **end for**

## 4. Theoretical Analysis

In this section, we present a theoretical analysis of the proposed network, focusing on its ability to mitigate domain shifts by extracting domain representations and modeling inter-domain relationships.

**Definition** **1.** *To facilitate the theoretical derivation, we represent the proposed neural network using formal mathematical notation. Let $f_{\theta }$ denote the encoder as $f_{\theta }$, with SA representing the self-attention mechanism, h representing the decoding network, and g representing the inference network. For any data (X,Y) in the query set of domain t, the prediction process can be formally represented by Equation (1). The self-attention mechanism can be interpreted as a weighted aggregation operation. Accordingly, the parameters of the inference network for domain t can be expressed as follows:*

(13) $$\begin{array}{c} \Phi_{t}^{\prime }=\frac{\sum_{s=1}^{T}d_{s,t}\Phi_{t}}{\sum_{s=1}^{T}d_{s,t}}, \end{array}$$

*where $d_{s,t}$ represents the similarity between domain s and t.*

**Definition** **2.** *We begin by assuming that the model has been fully trained on domains* 1 *through T. For the unseen test domain T+1, we employ a meta-learning strategy to extract its domain representation and apply the self-attention mechanism to integrate information from the previously seen domains. This process yields the inference network $g_{\Phi_{T+1}}$ for domain T+1. Given the ground-truth labels $Y_{T+1}$, we define the predictive or generalization error as $err:=l(g_{\Phi_{T+1}}\left(X_{t+1}\right),Y_{t+1})$, where l(.) denotes the loss function used for evaluation.*

**Definition** **3.** *To illustrate the impact of meta-learning on domain generalization, we introduce a comparison model. This model is trained on domains 1 through T; however, during testing on the unseen domain T+1, it does not utilize the support set $D_{T+1}^{su}$ to generate domain representations. Instead, it relies on the support set from the last training domain $D_{T}^{su}$ to construct the inference network, which is denoted as $g_{\Phi_{T}}$.*

**Theorem** **1.** *Following the prior works of Bai et al. [8], we assume $g_{\Phi_{T+1}}$ has a Lipschitz constant with upper bound $L_{\mathrm{upper}}$ and lower bound $L_{\mathrm{lower}}$; then, we have*

(14) $$\begin{array}{c} err\left(g_{\Phi_{T+1}}\right)<err\left(g_{\Phi_{T}}\right). \end{array}$$

*To prove the above theory, we use $q_{\theta }$ to represent the encoder parameterized by θ, and $P(\Phi_{1:T}|D_{1:T}^{Su})$ to denote the ideal posterior distribution. The process of optimizing the encoder can be seen as minimizing the gap between q and P, expressed as follows:*

(15) $$\begin{array}{c} \underset{\theta }{argmin}D_{KL}(q_{\theta }||P\left(\Phi_{1:T}|D_{1:T}^{Su}\right)). \end{array}\;$$

We assume that, initially, the encoder parameters are $\theta_{0}$, and, after optimization, the parameters become $\theta^{\prime }$, while the ideal parameters are denoted as $\theta^{*}$. We can assume that the optimized parameters are closer to the ideal parameters compared to the initial parameters, i.e.,

(16) $$\begin{array}{c} \left|\right|q_{\theta^{\prime }}\left(D_{T+1}^{Su}\right)-q_{\theta^{*}}\left(D_{T+1}^{Su}\right)\left||<|\right|q_{\theta_{0}}\left(D_{T+1}^{Su}\right)-q_{\theta^{*}}\left(D_{T+1}^{Su}\right)\left|\right|. \end{array}$$

Assuming Lipschitz continuity, let $\Phi^{*}$ denote the parameters of the ideal inference network. Then, the inference network satisfies the following property:

(17) $$\begin{array}{cc} \left|\right|g_{\Phi_{T}}\left(X\right)-g_{\Phi^{*}}\left(X\right)\left|\right| & >L_{lower}\cdot \left|\right|\Phi_{T}-\Phi^{*}\left|\right|,\\ \left|\right|g_{\Phi_{T+1}}\left(X\right)-g_{\Phi^{*}}\left(X\right)\left|\right| & <L_{upper}\cdot \left|\right|\Phi_{T+1}-\Phi^{*}\left|\right|. \end{array}$$

Based on Equation (14), we can assume that $\Phi_{T+1}$ is closer to the ideal parameters than $\Phi_{T}$. Therefore, we have $\left|\right|\Phi_{T+1}-\Phi^{*}\left||<|\right|\Phi_{T}-\Phi^{*}\left|\right|$. Moreover, since upper bound $L_{upper}$ is greater than lower bound $L_{lower}$, we have the following:

(18) $$\begin{array}{c} \frac{L_{upper}}{L_{lower}}\cdot \frac{\left|\right|\Phi_{T+1}-\Phi^{*}\left|\right|}{\left|\right|\Phi_{T}-\Phi^{*}\left|\right|}<1. \end{array}$$

Based on the above formulas, we have $\left|\right|g_{\Phi_{T+1}}\left(X\right)-g_{\Phi^{*}}\left(X\right)||<||g_{\Phi_{T}}\left(X\right)-g_{\Phi^{*}}\left(X\right)\left|\right|$. Since $g_{\Phi^{*}}\left(X\right)$ represents the optimal inference network, it follows that the model leveraging $D_{T+1}^{Su}$ as the support set outperforms the model that relies on $D_{T}^{Su}$ as the support set [8].

**Definition** **4.** *Our proposed method helps improve the domain generalization capability of the model. For a domain t that has not been seen during the training process, we aim to minimize the loss between the outputs of the inference model $g_{\Phi_{t}}$ parameterized by $\Phi_{t}$ and the true values. In addition, if the domain t is known, we can use traditional empirical risk minimization methods to train an inference model $g_{{\hat{\Phi }}_{t}}$ with parameter ${\hat{\Phi }}_{t}$. The closer $\Phi_{t}$ is to ${\hat{\Phi }}_{t}$, the better the performance of $g_{\Phi_{t}}$ is. Here, we assume that the domain relationship extraction module has been fully trained, such that $E[\left(g_{\Phi_{t}}\left(x\right)-g_{{\hat{\Phi }}_{t}}{\left(x\right)}^{2}\right]=O\left(\frac{C(H)}{n_{d}}\right)$, where C(H) is the Rademacher complexity of the function class H [14]. Then, we have the following theorem:*

**Theorem** **2.** *Suppose the number of meta-testing data for training domain d is $n_{d}$. If the loss function is Lipschitz continuous, then for the unseen test domain T+1, the excess risk satisfies [14]:*

(19) $$\begin{array}{cc} E_{\left(x,y\right)∼P_{T+1}}\left[L\left(g_{\Phi_{T+1}}\left(x\right),y\right)\right]-E_{\left(x,y\right)∼P_{T+1}}\left[L\left(g_{{\hat{\Phi }}_{T+1}}\left(x\right),y\right)\right]\leq B & +\sqrt{\frac{C\left(H\right)/n}{N^{tr}B^{r}}}, \end{array}$$

*where $N^{tr}$ represents the number of training domains. Theorem 2 suggests that during the training phase, increasing the number of domains improves the performance of $g_{\Phi_{T+1}}\left(x\right)$.*

## 5. Experimental Evaluation

### 5.1. Datasets Descriptions

To evaluate the performance of our model on time series prediction of sensor data, we conducted experiments on three traffic sensor datasets: PEMS04, PEMS07, and PEMS08 (The traffic sensor datasets provided by the authors of STID, https://drive.google.com/drive/folders/14EJVODCU48fGK0FkyeVom_9lETh80Yjp (accessed on 14 July 2025)). These datasets contain traffic flow data collected from California highways, with multiple sensors recording data every 5 min. PEMS04 includes 307 sensors over 59 days, PEMS07 includes 883 sensors over 98 days, and PEMS08 includes 170 sensors over 61 days. Each dataset also provides a graph representing the connectivity between sensor locations. For each dataset, we used the past 60 min of data (corresponding to a sequence length of 12 time steps) to predict traffic flow for the next 15, 30, and 60 min, which correspond to prediction horizons of 3, 6, and 12 time steps, respectively. We assumed that data collected within each day share the same distribution. Each dataset was split into training, validation, and test sets with proportions of 60%, 20%, and 20%, respectively.

The sensors in the above three datasets have spatial relationships. To evaluate the performance of our method on sensors without spatial relationships, we conducted experiments on the ETTh1, ETTh2 and Weather datasets (The time series datasets provided by the authors of DLinear, https://drive.google.com/drive/folders/1ZOYpTUa82_jCcxIdTmyr0LXQfvaM9vIy (accessed on 14 July 2025)). The ETTh1 and ETTh2 datasets monitor the operation of electrical transformers in a province in China, with a sampling frequency of one hour. Each data point includes the target variable “oil temperature” along with six electrical load features. The Weather dataset contains data collected from the Max Planck Institute for Biogeochemistry (Jena, Germany), including 21 meteorological parameters such as air temperature, humidity, and pressure. For each dataset, we used 96 time steps as input to predict the next 96, 192, 336, 720 time steps. Each dataset was split into training, validation, and test sets, with proportions of 60%, 20%, and 20%.

### 5.2. Baselines

For the traffic sensor datasets, we included two traditional statistical forecasting methods as baselines: HI (Historical Inertia) [34], which directly uses the most recent observation as the prediction, and VAR (Vector AutoRegression) [35], a classical multivariate time series model that captures linear dependencies among sensor nodes over time. In addition, we selected six state-of-the-art models for comparison: DCRNN [24], which uses a bidirectional diffusion process for the first time to capture spatial relationships between sensors; DGCRN [36], which employs a generated hypergraph to represent sensor connections; STID [37], which uses an MLP-based approach to embed spatiotemporal information into vectors for prediction; STAEformer [38], which utilizes a transformer to encode time series data; and GMMLP [39], which leverages a memory network to retain temporal sequence information.

For sensor datasets without spatial relationships, we also included HI and VAR as traditional baselines, and we further selected six state-of-the-art models for comparison in the field of time series forecasting: DRAIN [8], a temporal domain generalization method; MSGNet [3], which leverages frequency domain analysis and adaptive graph convolution; TimesNet [40], based on periodic decomposition; DLinear [22], which employs linear layers; and FEDformer [20] and Informer [1], both Transformer-based architectures.

### 5.3. Implementation Details

We used the Adam optimizer to train our model. During both training and testing, the batch size was set to 32, and the learning rate was set to 0.001. All models were trained for a maximum of 100 epochs, with early stopping applied if validation loss did not improve for 10 consecutive epochs. For the DCGRU-based encoder, we followed the setup by Li [24], using a two-layer GRU structure with 64 units per layer. The maximum diffusion steps K were set to 3. For the LSTM-based encoder used with non-spatial structured datasets, we used a two-layer LSTM structure with 64 units per layer. The latent domain representation z was set to a 64-dimensional vector. The self-attention module used for modeling inter-domain relationships was implemented as a Transformer encoder with two layers and two attention heads per layer. After passing through the self-attention mechanism, the decoder generates outputs through a multilayer perceptron (MLP) consisting of two layers. Furthermore, the parameter λ in the loss function was set to 0.1. For evaluation, we reported the average results across five runs using different random seeds. All experiments were conducted on an NVIDIA 24GB 4090D GPU (Nvidia, Santa Clara, CA, USA).

### 5.4. Results and Analysis

**Prediction Results**. Table 1 summarizes the performance of our method compared to other approaches across the three datasets. We evaluated using three metrics: MAE, RMSE, and MAPE. The best results for each metric are highlighted in bold. The results show that our model outperforms the latest state-of-the-art method, GMMLP, on the PEMS07 and PEMS08 datasets. On the PEMS04 dataset, our model achieved the best performance for prediction horizons of 3 and 6 time steps, though it was slightly outperformed by previous models at the 12-step horizon.

**Figure 2:**
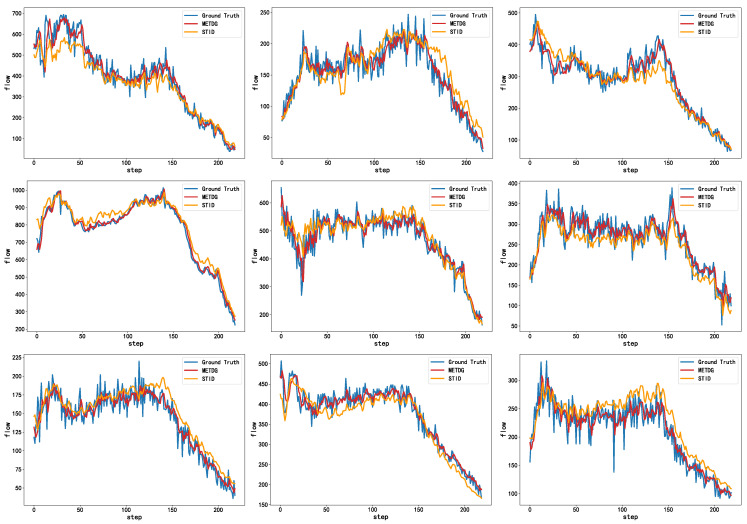
A visual comparison of the METDG and STID models on the PEMS04, PEMS07, and PEMS08 datasets. The top three images show the prediction results for sensors 75, 133, and 300 on the PEMS04 dataset. The middle three images represent the prediction results for sensors 13, 443, and 836 on the PEMS07 dataset. The bottom three images show the prediction results for sensors 8, 77, and 96 on the PEMS08 dataset.

**Figure 3:**
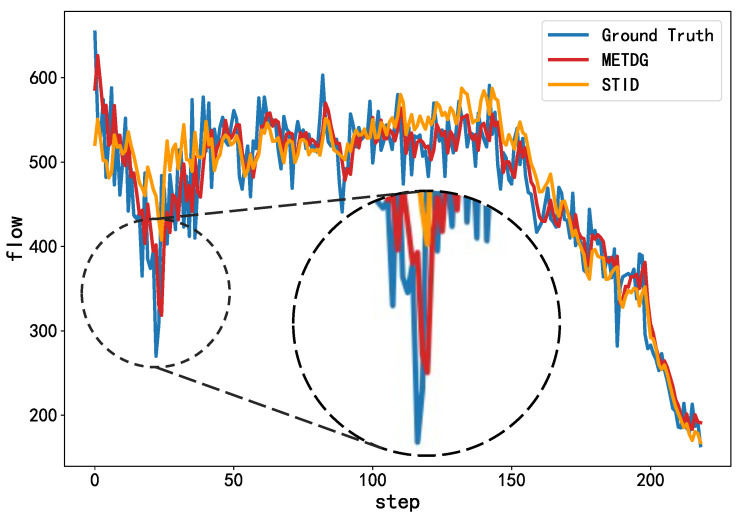
Zoomed-in view of a non-stationary period for Sensor 443 in the PEMS07 dataset.

We present the forecasting results using the traffic dataset in Figure 2 to showcase the overall predictive performance of our model. It can be seen that our method effectively captures both the global trend and local fluctuations in the time series, outperforming the baseline model. In addition, to emphasize the robustness of the model under non-stationary conditions, we provide a zoomed-in view of a segment for Sensor 443 in the PEMS07 dataset with rapid changes. As shown in Figure 3, our model exhibits stronger adaptability to sudden variations, providing more accurate predictions, whereas the baseline model tends to oversmooth the outputs.

Table 2 shows the performance of our method on three sensor datasets without spatial relationships. We used MSE and MAE as evaluation metrics. The best results for each metric are highlighted in bold. As can be seen, on the Weather dataset, our method achieves the best performance for 96-step and 192-step forecasting, while MSGNet outperforms it at 336 and 720 steps. On the ETTh1 dataset, our method achieves the best results at 96, 192, and 336 steps, but is outperformed by MSGNet at 720 steps. On the ETTh2 dataset, our method achieves the best performance across all metrics.

**Figure 4:**
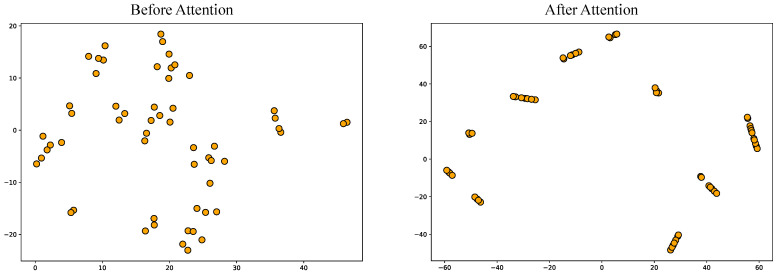
Domain representations after dimensionality reduction using the T-SNE algorithm. The left plot illustrates the representations before domain relationship modeling, while the right plot shows the representations after domain relationship modeling.

**Model Parameter Relationship Analysis**. To validate the effectiveness of our proposed method in capturing domain relationships, we conducted experiments on the PEMS04 dataset. First, domain representations were extracted using the encoder, resulting in domain representation vectors. Next, domain relationships were modeled using a self-attention mechanism to produce domain relationship vectors. These vectors were then reduced to two dimensions via the t-SNE algorithm and visualized on a two-dimensional plane. A comparison of the domain relationship distributions is shown in Figure 4. Notably, the distribution of domain representations changes markedly after incorporating the domain relationship weighting. Prior to applying the self-attention mechanism, the representation vectors were dispersed relatively evenly across the plane. After modeling the domain relationships, the vectors exhibited clear clustering patterns. This demonstrates that the self-attention mechanism effectively captures and models relationships between domains, grouping together domains with similar characteristics.

**Ablation Study.** To validate the effectiveness of our proposed method, we conducted ablation experiments on the PEMS04 dataset. The key innovations of our approach lie in modeling domain relationships via the self-attention mechanism and extracting domain representations using variational inference. To assess the impact of these components, we performed ablation by selectively removing them from the model. The experimental configurations are as follows:

1. **w/o Attention**: This variant excludes the self-attention mechanism. Domain features are first extracted using the DCGRU-based encoder, but the relationships between domains are not modeled with self-attention. Instead, the representation of each domain is directly used to generate the parameters of the corresponding inference network.
2. **w/o Variation**: This variant omits variational inference. During training, the variational lower bound is not employed as a loss term to regularize the domain representation generation process.
3. **w/o Attention +V ariation**: In this variant, both the self-attention mechanism and variational inference are removed. The inference network parameters are generated directly from domain representations, and the variational lower bound is excluded from the loss function.

**Figure 5:**
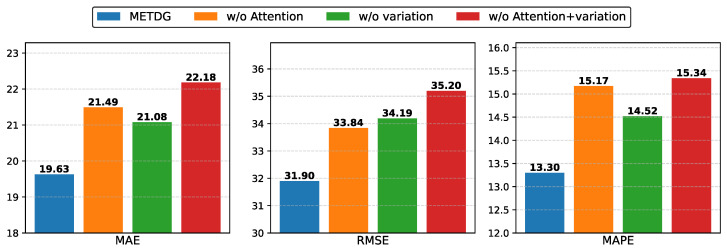
The results of the ablation study on PEMS04.

The ablation experiment results, shown in Figure 5, demonstrate that removing the self-attention mechanism and the variational lower bound significantly degrades model performance across all evaluation metrics—MAE, RMSE, and MAPE. The self-attention mechanism effectively captures similarity relationships between domains, enabling the inference network parameters to leverage information from related domains. Meanwhile, the variational lower bound acts as a constraint during domain representation generation, ensuring that the learned representations more accurately correspond to their respective domains.

**Figure 6:**
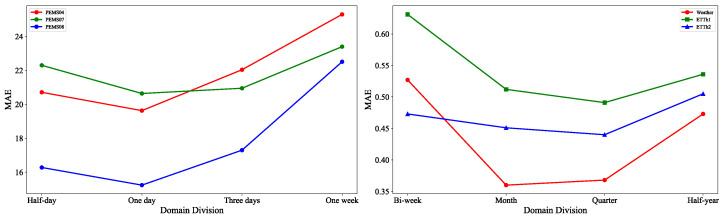
The impact of different domain divisions on the prediction results.

**Hyper-Parameters Analysis.** In our work, there is a fundamental assumption that the data within the same time segment follow the same distribution and the domain division is performed manually based on empirical knowledge or some predefined rules. To investigate the impact of different division ways on the results, we conducted experiments on both a traffic sensor datasets and a sensor datasets without an explicit graph structure. For the traffic datasets, the domains were divided by half a day, one day, three days, and one week. For the ETTh1, ETTh2, and Weather datasets, we segmented the domains on bi-weekly, monthly, quarterly, and half-yearly scales. The experimental results are shown on the left and right sides of Figure 6, respectively.

As shown in the figure, dividing each day into a separate domain achieved the best performance for the three traffic sensor datasets. For the ETTh1 and ETTh2 datasets, dividing the data by quarters yielded the best results, while for the Weather dataset, monthly segmentation was more effective. If the segmentation span is too small, it can lead to overfitting; if the span is too large, the data distribution within the segments may differ significantly, which can also degrade the model’s performance.

**Figure 7:**
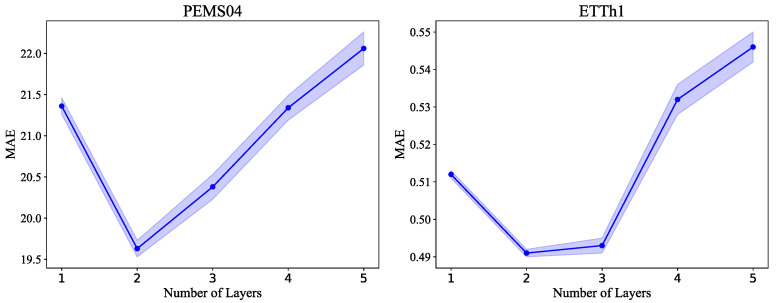
The impact of different depths of inference network on model performance. The blue dots indicates the mean value, and the blue shaded area represents the standard error range.

In addition, we examine how the number of layers in the inference model affects performance. We selected the PEMS04 and ETTh2 dataset, and set the number of layers in the inference network between one and five. The MAE curves are shown in Figure 7. As can be seen, for both datasets, setting the inference network to two layers yielded the best results. If the number of layers is too small, the network cannot effectively extract information from historical data. If the number of layers is too large, the randomness in parameter generation increases the inference network’s error, thereby reducing its inference capability.

**Figure 8:**
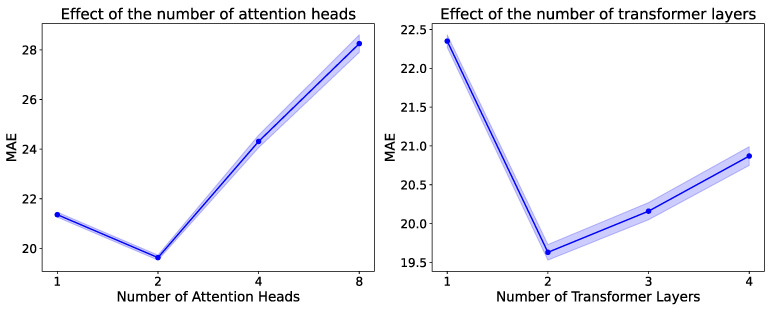
The impact of attention heads and transformer layers on model performance. The blue dots indicates the mean value, and the blue shaded area represents the standard error range.

Finally, we conducted a set of controlled experiments on the PEMS04 dataset to analyze the impact of two key hyperparameters in our model: the number of attention heads and the number of Transformer encoder layers. The results are summarized in Figure 8. As shown in the left subfigure, setting the number of attention heads to two achieved the best forecasting performance. This can be attributed to the fact that two heads strike a balance between expressiveness and stability. One head may be insufficient to capture diverse inter-domain patterns, while using too many heads introduces redundancy and noise, potentially leading to overfitting or unstable training. Similarly, the right-hand side of the figure shows that setting the Transformer encoder depth to two layers yields the best results. A shallow encoder (e.g., one layer) may fail to model the complex dependencies among temporal domains, whereas deeper encoders tend to suffer from performance degradation due to overfitting or vanishing gradients, especially when the number of tasks is limited. These findings validate that a lightweight attention mechanism with moderate complexity is optimal for capturing global task relationships within our temporal domain generalization framework.

## 6. Conclusions

In this paper, we introduced METDG, an ensemble-based method for temporal domain generation focused on time series forecasting tasks. Our approach utilizes a meta-learning mechanism to capture diverse task contexts and generate task-specific model parameters. Additionally, it employs Transformers to automatically extract the relationships between tasks and adjust model parameters while accounting for the global temporal dynamics of the models. We evaluated the effectiveness of METDG across various datasets and consistently observed that it outperforms existing methods. One limitation is that the selection of segment lengths is based on predefined rules or empirical experience. In the future, we aim to introduce adaptive learning strategies, for example, those based on frequency characteristics.

## Figures and Tables

**Table 1:** Comparison of the results for the the sensor datasets with spatial relationships.

PEMS04	Horizon 3			Horizon 6			Horizon 12		
	MAE	RMSE	MAPE	MAE	RMSE	MAPE	MAE	RMSE	MAPE
HI	42.33	61.64	29.90%	42.35	61.66	29.92%	42.37	61.67	29.92%
VAR	21.94	34.30	16.42%	23.72	36.58	18.02%	26.76	40.28	20.94%
DCRNN	18.53	29.44	12.76%	19.79	31.3	13.57%	21.73	33.98	15.12%
DGCRN	17.98	29.08	12.20%	18.99	30.9	12.86%	20.44	33.23	13.87%
STID	17.51	28.48	12.00%	18.29	29.86	12.46%	19.58	31.79	13.38%
DRAIN	19.57	30.74	13.37%	21.84	33.62	14.27%	23.22	35.32	15.28%
STAEformer	17.47	28.9	11.94%	18.20	30.31	12.35%	**19.30**	32.10	**13.19%**
GMMLP	17.39	28.41	12.03%	18.18	29.82	12.34%	19.37	**31.57**	13.58%
Ours	**17.17**	**28.32**	**11.43%**	**17.97**	**29.68**	**12.25%**	19.63	31.90	13.30%
PEMS07	Horizon 3			Horizon 6			Horizon 12		
	MAE	RMSE	MAPE	MAE	RMSE	MAPE	MAE	RMSE	MAPE
HI	49.02	71.16	22.73%	49.03	71.18	22.75%	49.06	71.20	22.79%
VAR	32.02	48.83	18.30%	35.18	52.91	20.54%	38.37	56.82	22.04%
DCRNN	19.75	31.74	8.31%	21.6	35.12	9.00%	24.76	40.75	10.39%
DGCRN	18.43	30.97	8.76%	20.43	34.12	8.69%	21.44	37.93	9.91%
STID	18.37	30.39	7.74%	19.66	32.82	8.28%	21.53	36.04	9.13%
DRAIN	21.23	34.18	9.02%	23.23	37.71	10.14%	27.29	44.24	13.26%
STAEformer	18.09	30.15	7.56%	19.38	32.99	8.97%	21.41	35.99	8.97%
GMMLP	17.98	**30.05**	7.67%	19.24	32.66	8.08%	20.89	35.76	8.77%
Ours	**17.87**	30.13	**7.54%**	**19.17**	**32.40**	**7.96%**	**20.64**	**35.70**	**8.69%**
PEMS08	Horizon 3			Horizon 6			Horizon 12		
	MAE	RMSE	MAPE	MAE	RMSE	MAPE	MAE	RMSE	MAPE
HI	34.55	50.41	21.60%	34.57	50.43	21.63%	34.59	50.44	21.68%
VAR	19.52	29.73	12.54%	22.25	30.30	14.23%	26.17	38.97	17.32%
DCRNN	15.64	25.48	10.04%	17.88	27.63	11.38%	22.51	34.21	14.17%
DGCRN	13.89	22.07	9.19%	14.92	23.99	9.85%	16.73	26.88	10.84%
STID	13.85	21.92	9.03%	15.00	24.04	9.78%	16.77	26.91	10.93%
DRAIN	16.88	26.73	11.13%	18.86	30.17	13.22%	23.24	36.16	14.84%
STAEformer	13.85	21.99	8.76%	14.41	23.72	9.26%	15.34	26.03	9.91%
GMMLP	13.18	21.62	8.55%	14.09	23.59	9.18%	15.32	26.06	9.90%
Ours	**13.12**	**21.53**	**8.50%**	**13.99**	**23.24**	**9.01%**	**15.24**	**25.89**	**9.85%**

**Table 2:** Comparison of results for the sensor datasets without spatial relationships.

Methods		Ours		HI		VAR		DRAIN		MSGNet		Timesnet		DLiner		FEDformer		Informer	
Metric		MSE	MAE	MSE	MAE	MSE	MAE	MSE	MAE	MSE	MAE	MSE	MAE	MSE	MAE	MSE	MAE	MSE	MAE
Weather	96	**0.160**	**0.199**	0.894	0.553	0.458	0.491	0.236	0.278	0.163	0.212	0.220	0.196	0.196	0.255	0.217	0.296	0.300	0.384
	192	**0.207**	**0.244**	0.621	0.628	0.648	0.580	0.279	0.301	0.212	0.254	0.219	0.261	0.237	0.296	0.276	0.336	0.598	0.544
	336	0.275	**0.294**	0.736	0.753	0.797	0.659	0.329	0.365	**0.272**	0.299	0.280	0.306	0.283	0.355	0.339	0.380	0.578	0.523
	720	0.364	0.360	1.005	0.934	0.869	0.672	0.379	0.428	**0.350**	**0.348**	0.365	0.359	0.345	0.381	0.403	0.428	1.059	0.741
ETTh1	96	**0.372**	**0.395**	1.264	0.934	0.871	0.749	0.440	0.451	0.390	0.411	0.384	0.402	0.386	0.400	0.376	0.419	0.865	0.713
	192	**0.420**	**0.432**	1.457	0.980	1.554	1.317	0.490	0.482	0.442	0.442	0.436	0.429	0.437	0.432	0.420	0.448	1.008	0.792
	336	**0.454**	**0.457**	2.025	1.138	1.420	1.128	0.520	0.496	0.480	0.468	0.491	0.469	0.481	0.459	0.459	0.465	1.107	0.809
	720	0.510	0.491	2.383	1.376	1.937	1.322	0.511	0.502	**0.494**	**0.488**	0.521	0.500	0.519	0.516	0.506	0.507	1.181	0.865
ETTh2	96	**0.311**	**0.330**	2.576	1.148	1.539	1.618	0.346	0.388	0.328	0.371	0.340	0.374	0.333	0.387	0.358	0.397	3.755	1.525
	192	**0.329**	**0.347**	3.338	1.783	2.879	1.534	0.392	0.407	0.402	0.414	0.402	0.414	0.477	0.476	0.429	0.439	5.602	1.931
	336	**0.350**	**0.390**	2.936	1.780	3.452	1.693	0.421	0.434	0.435	0.443	0.452	0.452	0.594	0.541	0.496	0.487	4.721	1.835
	720	**0.385**	**0.440**	4.625	3.821	5.385	2.097	0.452	0.473	0.417	0.441	0.462	0.468	0.831	0.657	0.463	0.474	3.647	1.625

## Data Availability

The data that support the findings of this study come from public resources.
